# Blue Marble Health and the Global Burden of Disease Study 2013

**DOI:** 10.1371/journal.pntd.0004744

**Published:** 2016-10-27

**Authors:** Peter J Hotez, Ashish Damania, Mohsen Naghavi

**Affiliations:** 1 Sabin Vaccine Institute and Texas Children’s Hospital Center for Vaccine Development, National School of Tropical Medicine at Baylor College of Medicine, Houston, Texas, USA; 2 Department of Biology, Baylor University, Waco, Texas, USA; 3 Center for Health and Biosciences, James A Baker III Institute for Public Policy, Rice University, Houston, Texas, USA; 4 Institute for Health Metrics and Evaluation, University of Washington, Seattle, Washington, USA

The concept of blue marble health emerged as a novel framework for global health in 2013 [1, 2]. Succinctly put, today most or at least one-half of the world’s neglected diseases occur among the poor living in wealthy countries, especially in the group of 20 (G20) nations and Nigeria [1–4]. Based on data mostly compiled and released by the WHO (and other published sources), approximately one-half of the major helminth infections occur among the G20 countries and Nigeria, as well as most of the dengue, leishmaniasis, leprosy, Chagas disease, and possibly other neglected tropical diseases (NTDs) [1–3]. In addition, most (57%) of the tuberculosis (TB) cases are found in these countries, as are almost one-half of the malaria (45%) and HIV/AIDS (44%) cases [4].

The term “blue marble” is based on a famous Earth photograph taken by astronauts from an Apollo mission, which has since become an important symbol for the health of our planet [5]. We sometimes refer to the G20 nations and Nigeria as the blue marble health countries.

An important policy implication of blue marble health is that because these neglected diseases are endemic to wealthy nations with high gross domestic products (GDPs), in many cases the resources to combat these diseases through treatment and prevention measures, as well as resources for research and development for new technologies, should be available [6]. By taking greater ownership of their own public health control and policy efforts, the blue marble health countries could eliminate at least one-half of the world’s neglected diseases [6]. In addition, we found that the blue marble health countries account for approximately 70% of the deaths from noncommunicable diseases [7].

**Fig 1:**
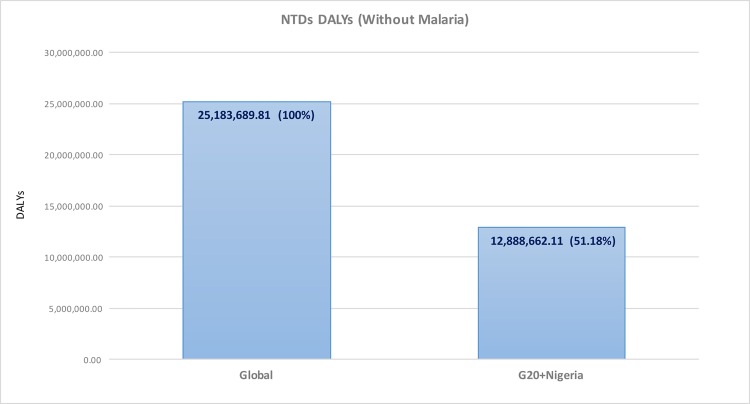
Comparison of DALYs due to NTDs in the G20 countries and Nigeria compared to global NTD DALYs. Original figure based on data from IHME. [8]

**Fig 2:**
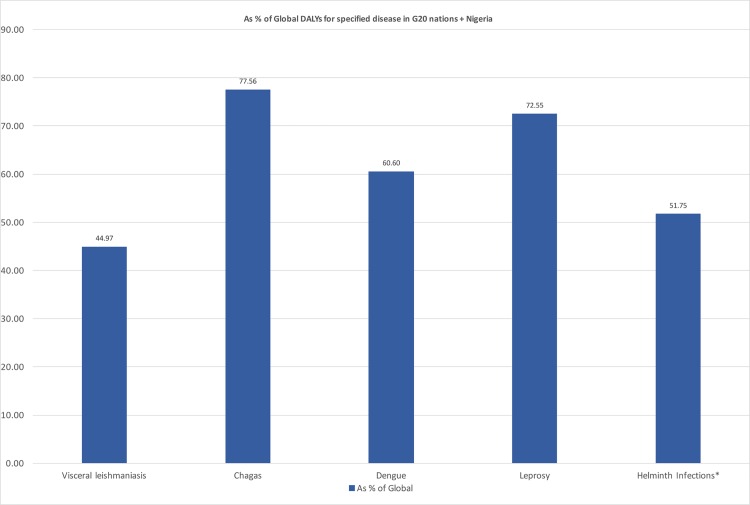
Percentage of the DALYs of the major NTDs found in the G20 countries and Nigeria. Original figure based on data from the Institute for Health Metrics and Evaluation (IHME) [8]. * Helminth Infections: Lymphatic Filariasis + Food-borne trematodiases + Cysticercosis + Cystic Echinococcosis + Onchocerciasis + Schistosomiasis + Ascariasis + Trichuriasis + Hookworm Disease.

**Fig 3:**
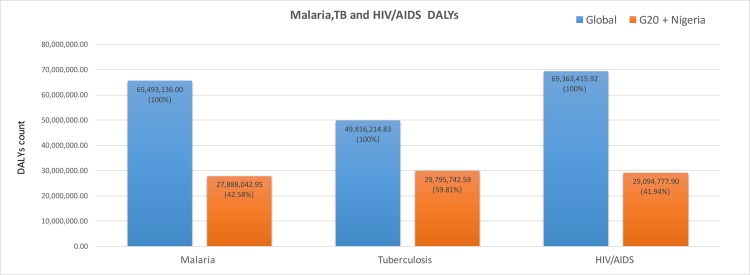
Comparison of DALYs due to malaria, tuberculosis (TB), and HIV/AIDS in the G20 countries and Nigeria (orange) compared to global malaria, TB, and HIV/AIDS DALYs (blue). Original figure based on data from IHME. [8]

Shown in Figs 1–3 is an analysis of data from the GBD 2013 that focused on the disability-adjusted life years (DALYs) due to NTDs and HIV/AIDS, TB, and malaria [8]. The GBD 2013 largely confirms the conclusions of the WHO and other data analyzed previously. Thus, the G20 nations and Nigeria together account for 51% of the global DALYs due to NTDs and helminth infections and most of the DALYs resulting from Chagas disease, dengue, and leprosy (Figs 1 and 2).

In addition, the blue marble health countries account for approximately 60% of the global TB DALYs and 43% and 42% of the malaria and HIV/AIDS DALYs, respectively (Fig 3).

Shown in Table 1 is a comparison of the estimates based on either WHO (and other) data and GBD 2013 data. It is interesting to note that for the helminth infections, for example, despite the fact that the WHO PCT data evaluates children or children and adults at risk who require mass drug administration, whereas the GBD 2013 estimates DALYs from actual infections, the two estimates of the percentage found in G20 countries and Nigeria are almost the same. Indeed, with the exception of visceral leishmaniasis, the results show a relatively high concurrence between GBD 2013 and WHO or other estimates, in terms of the high percentage of NTDs, HIV/AIDS, TB, and malaria found in G20 countries together with Nigeria. The basis for the different results for visceral leishmaniasis is under investigation, while the fact that the dengue estimates are identical between GBD 2013 and those reported earlier based on studies conducted by Bhatt et al. may reflect their use of similar or identical sources of data [9].

**Table 1:** Comparison of WHO (and other sources) and GBD 2013 disease burden estimates in terms of percentage in G20 countries^a^ and Nigeria. WHO and other sources data from references [2–4].

Source of Data	Percentage of NTDs in G20 + Nigeria	Percentage of HIV/AIDS in G20 + Nigeria	Percentage of Tuberculosis (TB) in G20 + Nigeria	Percentage of Malaria in G20 + Nigeria
WHO and Other Published Sources	50% Helminth infections, 61% dengue fever, 61% Chagas disease, 67% visceral leishmaniasis, and 77%–78% leprosy	44%	57%	45%
GBD 2013	52% Helminth infections, 61% dengue fever, 78% Chagas disease, 45% visceral leishmaniasis, and 73% leprosy	42%	60%	43%

^a^ The G20 nations include 19 countries—Argentina, Australia, Brazil, Canada, China, France, Germany, India, Indonesia, Italy, Japan, Mexico, Russia, Saudi Arabia, South Africa, South Korea, Turkey, the United Kingdom, and the United States of America—in addition to the European Union.

**Fig 4:**
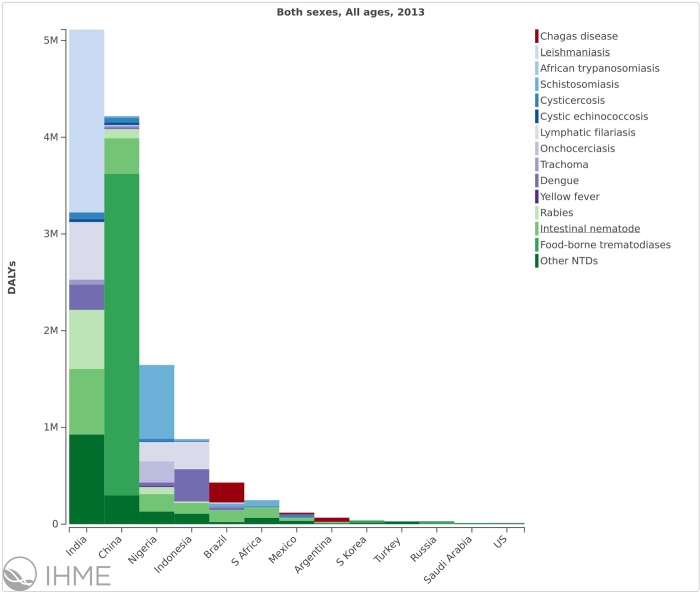
The DALYs from NTDs in the blue marble health countries. The figure was generated from the GBD Compare website: IHME. GBD Compare. Seattle, Washington: IHME, University of Washington, 2015. Available from http://vizhub.healthdata.org/gbd-compare. Accessed 3 January 2016. [8]

A further analysis of the NTD DALYs in the blue marble health countries is shown in Fig 4. It shows that India has the highest disease burden, led by leishmaniasis, followed by China, mostly due to food-borne trematodiases. Nigeria exhibited the third highest NTD burden, followed by Indonesia, Brazil, South Africa, Mexico, Argentina, South Korea, Turkey, and Russia. However, the disease burdens for some blue marble health countries may be underestimated in the GBD 2013. For example, the US has a substantial burden of Chagas disease and cysticercosis that is not represented in the GBD 2013, while for other neglected diseases that are widespread in the US, such as toxoplasmosis and toxocariasis, there are no specific DALYs assigned in either the GBD 2010 or GBD 2013 [8].

**Fig 5:**
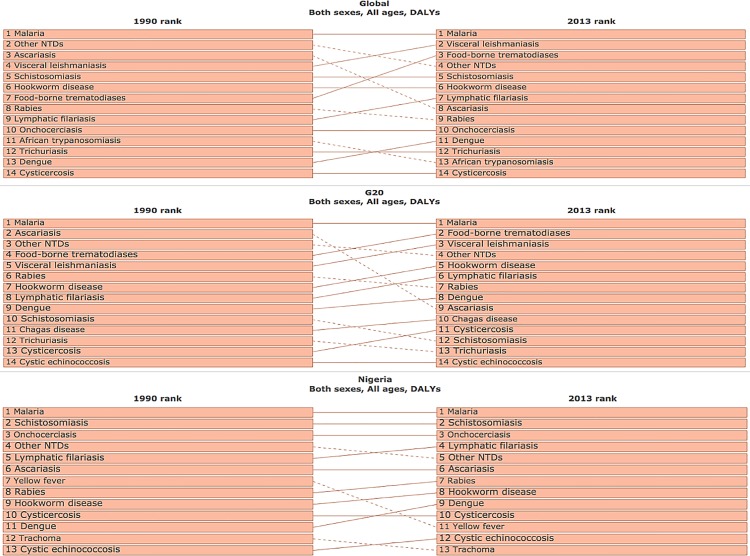
NTDs ranked in terms of DALY count. The figure was generated from the GBD Compare website: IHME. GBD Compare. Seattle, WA: IHME, University of Washington, 2015. Available from http://vizhub.healthdata.org/gbd-compare. Accessed 3 January 2016. [8]

Finally, Fig 5 shows some recent changes in the ranking of the NTDs and malaria in the G20 countries and Nigeria since 1990. Regarding the G20 countries, ascariasis exhibited the greatest drop in rank, possibly due to mass drug administration using anthelminthic drugs, whereas the food-borne trematodiases, visceral leishmaniasis, hookworm, lymphatic filariasis, dengue, Chagas disease, and cysticercosis went up in rank. For Nigeria there appeared to be no major shifts in the rankings since 1990 [8].

In summary, information using DALYs from the GBD 2013 confirms the findings related to blue marble health that were previously derived using WHO and other data. The concurrence provides further impetus for pursuing public policies related to a framework for blue marble health for the G20 countries and Nigeria. Among those policies is greater engagement by G20 government leaders to provide mass drug administration for the major NTDs affecting their vulnerable populations, in addition to preventive measures for HIV/AIDS, TB, and malaria [6]. There is also heightened urgency to increase commitments for neglected diseases research and development (R&D) among the G20 leaders [6].
